# Gambling and public health: we need policy action to prevent harm

**DOI:** 10.1136/bmj.l1807

**Published:** 2019-05-08

**Authors:** Heather Wardle, Gerda Reith, Erika Langham, Robert D Rogers

**Affiliations:** 1 London School of Hygiene and Tropical Medicine, 15-17 Tavistock Place London, London WC1E 7HT, UK; 2 University of Glasgow College of Social Sciences, Glasgow, Glasgow, UK; 3 Central Queensland University School of Human Health and Social Sciences, Cairns, Queensland, Australia; 4 Bangor University College of Health and Behavioural Sciences, Bangor, Gwynedd, UK

## Abstract

Prevention of harms related to gambling requires investment in population based approaches, say **Heather Wardle and colleagues**

**Key messages**

Current approaches targeting affected individuals substantially underestimate the harms of gambling
Gambling places a major burden of harm on individuals, communities, and society
Harms from gambling are generated through a range of political, legislative, commercial and interpersonal actions
Public health approaches to reduce harms related to gambling should encompass a range of population based approaches supported by regulation, legislation. and funding

In 2017 the gambling regulator for Great Britain, the Gambling Commission, described problem gambling as a public health concern (box 1)^3^ and emphasised the need to increase protection from harm.^4^ In 2018 the Faculty of Public Health released a position paper arguing for the introduction of harm prevention measures, underpinned by legislation, targeted at the whole population.^5^ The Labour Party recently shared plans for a radical overhaul of legislation to reduce the harms associated with “Britain’s hidden epidemic.”^6^

Box 1 **Gambling behaviour in Great Britain^1^ ^2^**

Gambling encompasses a broad range of activities, ranging from the National Lottery to casino games, slot machines, and online betting
Around 58% of adults in Great Britain gambled on at least one of these activities in the past year
Approximately 0.7% of adults (about 340 000 people) in Great Britain are problem gamblers and a further 1.1% (about 550 000) are at moderate risk of harms related to gambling
Online gambling—on casino or slot style games and sports betting—is the largest growth area in the sector, accounting for over a third of the market. There are over 33 million active online gambling accounts in Great Britain
The prevalence of online gambling has increased from less than 1% in 1999 to 9% in 2016, with many online gamblers holding multiple accounts. This makes online gambling as popular as traditional betting on horses and more popular than playing slot machines or visiting casinos
14% of children aged 11-16 have gambled in the past week, with around 55 000 reporting problems from their gambling behaviour

Despite these announcements, commercial gambling in Great Britain, as in many other jurisdictions, is still not legislated as a public health problem. Simply stating that gambling is a public health concern is not enough. It must also be treated as one by policy makers through the development and implementation of a fully realised and sustainably funded strategy for preventing harms among the population.

## Understanding gambling related harms

The first step towards developing effective harm prevention policies lies in identifying the nature and scale of the issue. Until recently, the health effects of gambling were largely understood in terms of individual pathology, based on the categorisation of clinical symptoms or behaviours, such as preoccupation with gambling, failed attempts to stop, increasing tolerance for gambling or gambling to escape problems, using specified diagnostic criteria as set out in the American Psychiatric Association’s Diagnostic and Statistical Manual of Mental Disorders.^7^ But this perspective identifies only a small minority of the population as having gambling problems. This, together with neoliberal ideas of health promotion that emphasise individual responsibility for health choices,^8^ has focused policy attention on the treatment of a minority of “problem gamblers” and the promotion of “responsible gambling” and self control. This approach is supported and promoted by industry, a powerful actor in this system. As with discussions around other products harmful to public health, such as processed foods and alcohol,^9^ ^10^ focusing on the individual aligns with industry interests by shifting regulatory attention away from the products and commercial practices that generate harms and from the broader policy measures that would restrict and regulate their availability.

We need a systematic reframing of the issue that recognises the major burden of harms that gambling places on not only individuals but also communities and society^11^ ^12^ and that acknowledges the role of commercial, policy, and regulatory forces in shaping the environment in which these harms occur. Then we need a shift in policy that focuses on the broader effects of gambling on individuals, families, friends, communities, and society. These effects include financial problems, relationship breakdowns, abuse or neglect of partners and children, and adverse childhood experiences that disrupt relationships and education during periods of cognitive and social development.^13^

Harms related to gambling reflect social and health inequalities, with negative effects unequally distributed among economically and socially disadvantaged groups and are commonly associated with a range of mental and physical health comorbidities.^14^ ^15^ At its most severe, gambling can contribute to loss of life. Research from Victoria, Australia, estimated that around 2% of suicides between 2010 and 2012 were related to gambling.^16^

Broadening our focus beyond problem gambling reveals the true scale of its negative effects and has implications for estimating its economic and social costs. Harms affect a much larger proportion of the population than just those who might be defined as problem gamblers: for every one person with problems, an estimated five to 10 people are adversely affected.^17^ In Australia, the burden of harms that gambling places on health and wellbeing is estimated to be of similar magnitude to major depressive disorder or alcohol misuse and dependence.^11^ In Great Britain, conservative estimates of social costs range between £200m (€230m; $260m) and £1.2bn a year, and these are likely to be considerable underestimates.^18^

Epidemiological evidence indicates high levels of “churn” in and out of problematic and at-risk behaviour. In Britain, a follow-up study of highly engaged gamblers (individuals with loyalty cards for major bookmakers) showed that around one in three people defined as non-problem, low risk, or moderate risk (according to their scores on the Problem Gambling Severity Index) had increased their problem gambling scores when interviewed one year later.^19^ Longitudinal research in Australia found that the number of newly identified problem gamblers accounted for half of the prevalence rate, signifying high degrees of movement in and out of this kind of behaviour.^20^ Such volatility reinforces arguments for targeting resources towards harm prevention to avoid escalation.

Harms from gambling affect health and wellbeing and, even at low risk levels, contribute to a loss of quality of life similar to the long term consequences of a moderate stroke, moderate alcohol use disorder, and urinary incontinence.^11^ These low level harms arguably contribute more to aggregate social costs than those from people gambling at problematic levels because of the greater population numbers experiencing them. Australian research found that up to 85% of the harms caused by gambling came from those who were not categorised as problem gamblers.^11^ ^12^ This indicates that current calculations of the social costs of gambling in Britain, which focus only on costs generated by the small number of individuals categorised as problematic, are likely to be major underestimates. As such, there are likely to be considerable, but as yet unquantified, burdens placed on the health, welfare, and judicial systems dealing with the consequences of these harms.

A recent report for the Gambling Commission has drawn on the broader approaches newly adopted in Victoria, Australia, and New Zealand to produce a pragmatic definition of gambling related harms intended to guide policy formation (box 2).^21^

Box 2 **Definition of gambling related harms proposed by the Gambling Commission^13^**

Gambling related harms are the adverse impacts from gambling on the health and wellbeing of individuals, families, communities and society
These harms are diverse, affecting resources, relationships, and health, and may reflect an interplay between individual, family, and community processes. The harmful effects of gambling may be short lived but can persist, having longer term and enduring consequences that can exacerbate existing inequalities

## Broader understanding of the determinants of harms

**Fig 1:**
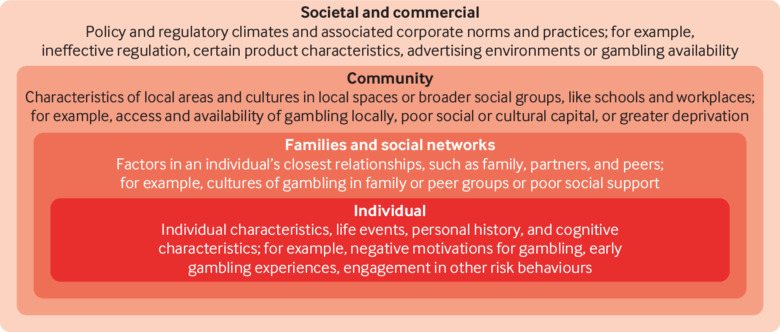
The social-ecological model for gambling. Factors that influence the potential experience of harm.

Shifting the focus away from harms as being generated by a small number of individuals who are experiencing a clinical disorder brings with it a reconsideration of the broader determinants of those harms. An interplay of individual, social, and environmental processes is known to contribute to many illnesses.^22^ Around 50% of global variation in health status is attributable to social and environmental context,^23^ and gambling is unlikely to be different. Those who gamble (harmfully or not) are embedded within an environment shaped by commercial, legislative, regulatory, and cultural forces that determine the availability and accessibility of gambling products and venues, as well as the advertising and promotion of gambling on a wide scale (fig 1). Since implementation of the Gambling Act 2005 the scale and sophistication of industry marketing has increased in both land based and online contexts.^24^ As with alcohol and unhealthy foods, commercial gambling is sustained and promoted by a powerful global industry in ways that not only make it more widespread but also shape how we think about appropriate policy responses to the health effects of its products.^9^

## Implications for policy

Recognising the wider environmental and commercial determinants of harm requires a re-orientation of policy and practice. Effective preventive action needs to go beyond existing interventions aimed at individuals, which have largely relied on industry led measures targeted at high risk individuals, for example through the development of algorithms to detect harmful levels of play (in online settings) or the voluntary setting of time and money limits. As a recent review notes, prevention activity in Britain has been underspecified and is inadequate.^25^

Activities targeted at high risk individuals certainly form part of a coherent prevention strategy, but we also need legislative or regulatory measures that tackle the availability, licensing, advertising, and price of products. Other public health contexts show how measures that affect the whole population (such as smoke-free legislation in Britain) often have the biggest effect on behaviour change.^26^ Such measures should be used to regulate the design, licensing, and placement of gambling products, such as high intensity, high volatility, or high stakes gambling machines, throughout communities. They could be used to restrict the use of credit to gamble online or introduce mandatory affordability checks. They should also be used to curtail the scale and scope of industry advertising and marketing, particularly personalised marketing, through legislation.

## Legislative and funding environment

Effective policy to reduce gambling related harms needs to adopt a broad focus, with strategic action planned and delivered to deal with the multifactorial determinants of health. This is well recognised for obesity, smoking, and alcohol consumption, but Britain has no government owned strategy for preventing harm from gambling.

British legislation currently seeks to balance enabling gambling with protecting (some) vulnerable people in a poorly specified way (box 3). Protecting vulnerable people from harm is a licensing requirement, but so too is “aiming to permit” gambling, and there is no guidance about the extent to which gambling could or should be curtailed in order to protect vulnerable groups. This contradiction needs to be tackled, and the protective mechanisms of the act strengthened.

Box 3 **Gambling legislation and policy in Great Britain:**

The Gambling Act 2005 updated gambling policy and legislation in Great Britain
The three licensing objectives in the act are:
  - preventing gambling from being a source of crime or disorder, being associated with crime or disorder, or being used to support crime
  - ensuring that gambling is conducted in a fair and open way
  - protecting children and other vulnerable people from being harmed or exploited by gambling
Gambling is be treated as a valid leisure and recreational choice, meaning it can be freely promoted (subject to some limitations on advertising) and that licensing authorities have to “aim to permit” gambling as long as it is consistent with the three licensing objectives
Policy responsibility for gambling has been held by the Department of Digital, Culture, Media, and Sport (DDCMS) since 2007
Until March 2019, the National Responsible Gambling Strategy was produced by the Responsible Gambling Strategy Board, an independent advisory group to the regulator (Gambling Commission). From April 2019, it will be owned by the regulator. Neither DDCMS nor any other government department has responsibility for the strategy
The National Responsible Gambling Strategy and its successor will continue to be funded through voluntary donations by industry unless a statutory levy on industry is invoked

In New Zealand, harm reduction is a legislative requirement, and the annual budget for the prevention of gambling harms is over $NZ18m (£9.3m; €10.7m; $12m) for a population of 4.7 million.^27^ ^28^ By contrast, in 2017-18 Britain had £8m for gambling research, education, and treatment for a population of 65 million; less than £1.5m was spent on prevention activity.^29^ In Britain, this funding relies on voluntary contributions from industry. The costs of gambling are likely to considerably outweigh the benefits (in terms of tax revenues), indicating that it actually costs societies more to not systematically address gambling harms.^12^ In Victoria, Australia, total tax revenue from gambling was $A1.6bn (£0.9bn; €1bn; $1.1bn) while estimated social costs were $AUS 6.97 billion, a net deficit of $AUS 5.4 billion.^12^

Funding for prevention and treatment of gambling related harms in Britain is woefully under-resourced, which needs urgent attention. The statutory power to impose a compulsory levy on industry exists, but successive governments have been unwilling to enact the levy. This is despite the industry regulator, their advisers, and even some industry actors themselves supporting a levy.^4^ ^30^ This highlights why the broader system in which gambling policies are created and legislated must be considered.

Current policy responsibility for gambling is held by the Department for Digital, Culture, Media, and Sport rather than the Department of Health and Social Care, confirming that gambling is not considered a public health issue in the current legislative framework. Recent announcements around changes in the maximum stake sizes on so-called fixed odds betting terminal machines showed the political power of the Treasury, with the announced reduction in stake counterbalanced with an increase in remote gaming tax duty to ensure that the policy was cost neutral in tax revenue terms.^31^ This multiplicity of governmental actors, each with divergent or conflicting aims, slows the resolution of policy formulation and enactment.

If gambling is to be taken seriously as a public health issue then policy responsibility for prevention and treatment should lie with the Department of Health and Social Care, with input from other departments who deal with the harms of gambling such as welfare, justice, and education. Local authorities should also play a significant role given their responsibility for local public health policies, though their range of actions are constrained by the current legislative framework. The role of the NHS in this system should also be considered. Britain currently has only one NHS clinic for the treatment of gambling problems, funded through a charitable organisation that disperses industry donations, though this exemplar shows how these clinics can be a catalyst for broader prevention and awareness raising activities.^32^ ^33^ The NHS long term plan, announced in January 2019, included commitments to expand the range of NHS treatment provisions for gambling, but what this means in practice and how it will be funded remain unclear.^34^

## Conclusions

Like other public health concerns, gambling is associated with wide ranging harms and disproportionately affects vulnerable groups in ways that contribute to and exacerbate existing social inequalities. It also imposes a large economic burden on society. The causes of harms are multifactorial, reflecting an interplay of individual, social, and environmental processes. Policy makers, especially those in central government, need to be aware of the potential health effects and substantial social costs of gambling and of the need to develop, fund, and implement strategies to prevent harm. These, crucially, should be evidence based and assessed for efficacy. In Britain, this policy does not yet exist, though the regulator is attempting to correct this. The policy and funding environment in which a coherent strategy for reducing gambling-related harms can be developed needs to be critically reassessed, along with the industry’s role in shaping existing practices. This requires a marked change in approach, and one that is long overdue, given that gambling harms are a matter of health equality and social justice.

## Notes

**Contributors and sources:** This paper has been partially funded by Wellcome through HW’s Wellcome Research Fellowship in Humanities and Social Sciences (grant number 200306/Z/15/Z). HW is deputy chair of the Advisory Board on Safer Gambling an independent group that provides advice to the Gambling Commission on policy and practice. GR is a Professor of Social Sciences at the University of Glasgow. RDR is a professor of psychology at Bangor University and EL is a lecturer health promotion at Central Queensland University, Australia. The genesis of this article came from two reports produced by three of the coauthors: one a review of gambling and gambling policy conducted for Public Health Wales by RDR, HW, and others^35^ and the other looking at the importance of measuring gambling related harms led by HW and GR.^13^ HW and GR acknowledge the contribution of pStephen Platt, David Best, and David McDaid to the harms report. Both reports draw on original work conducted by EL and colleagues, seeking to adapt and extend her research.^11^ All coauthors have contributed equally to the production of this manuscript and approved the final submission. HW is the guarantor for this article.

Provenance and peer review: Not commissioned; externally peer reviewed.

## References

[1] Connolly A Davies B Fuller L Heinze N Wardle H Gambling in Great Britain in 2016: evidence from England, Scotland and Wales. Gambling Commission, 2018.

[2] Gambling Commission Young people and gambling 2018. Gambling Commission, 2018.

[3] Miller T. Problem gambling: a public health issue. Speech given at the GambleAware Annual Conference, 2017. https://www.gamblingcommission.gov.uk/PDF/speeches/GambleAware-Conference-Tim-Miller-December-2017.pdf

[4] Gambling Commission Making gambling safer and fairer. 2017.

[5] Faculty for Public Health of the Royal Colleges of Physicians of the United Kingdom. Faculty of Public Health gambling policy statement. https://www.fph.org.uk/media/1810/fph-gambling-position-statement-june-2018.pdf.

[6] Labour Party. Labour party review of problem gambling and its treatment. https://d3n8a8pro7vhmx.cloudfront.net/campaigncountdown/pages/2214/attachments/original/1537438117/11519_18_Gambling_addiction_Paper-_Tom_Watson_v7_%28ELECTRONIC%29%28WEB%29.pdf?1537438117.

[7] American Psychiatric Association. Diagnostic and statistical manual of mental disorders DSM-IV-TR Fourth Edition (Text Revision). American Psychiatric Publishing, 2000.

[8] Hiam L Dorling D Government’s misplaced prevention agenda. BMJ 2018;363:k5134. 10.1136/bmj.k5134 30518515

[9] Moodie R Stuckler D Monteiro C Lancet NCD Action Group Profits and pandemics: prevention of harmful effects of tobacco, alcohol, and ultra-processed food and drink industries. Lancet 2013;381:670-9. 10.1016/S0140-6736(12)62089-3 23410611

[10] Jahiel RI Babor TF Industrial epidemics, public health advocacy and the alcohol industry: lessons from other fields. Addiction 2007;102:1335-9. 10.1111/j.1360-0443.2007.01900.x 17697267

[11] Browne M Langham E Rawat V Assessing gambling-related harm in Victoria: a public health perspective. Victorian Responsible Gambling Foundation, 2016.

[12] Browne M Greer N Armstrong T The social cost of gambling to Victoria. Victorian Responsible Gambling Foundation, 2017.

[13] Wardle H Reith G Best D McDaid D Platt S Measuring gambling-related harms: a framework for action. Gambling Commission, 2018.

[14] Cowlishaw S Kessler D Problem gambling in the UK. Implications for health, pyschosocial adjustment and healthcare utilisation. Eur Addict Res 2016;22:90-8. 10.1159/000437260 26343859

[15] Wardle H. Exploring area based vulnerability to gambling: who is vulnerable? City of Westminster, 2015.

[16] Coroners Court of Victoria. Coroner’s prevention unit: gambling related suicides, Victoria 2000-2012. 2016. http://www.coronerscourt.vic.gov.au/resources/64095dbc-321c-42e4-8f75-01a8d201c062/cpu+data+summary+-+gambling+related+suicides+-+10+sep+2013.pdf.

[17] Productivity Commission. Australia’s gambling industries. Report no 10. Australian government, 1999.

[18] Thorley C, Stirling A, Huynh E. Cards on the table—the cost to government associated with people who are problem gamblers in Britain. 2016. https://www.ippr.org/publications/cards-on-the-table

[19] Wardle H Fuller L Maplethorpe N Jones H Follow-up study of of loyalty card customers: changes in gambling behaviour over time. GambleAware, 2017.

[20] Billi R Stone CA Marden P Yeung K The Victorian gambling study: a longitudinal study of gambling and health in Victoria, 2008-2012. Victorian Responsible Gambling Foundation, 2014.

[21] Browne M Bellringer M Greer N Measuring the burden of gambling harm in New Zealand. New Zealand Ministry of Health, 2017.

[22] Krieger N Epidemiology and the people’s health: theory and context. Oxford University Press, 2011 10.1093/acprof:oso/9780195383874.001.0001 .

[23] Donkin A Goldblatt P Allen J Nathanson V Marmot M Global action on the social determinants of health. BMJ Glob Health 2017;3(Suppl 1):e000603. 10.1136/bmjgh-2017-000603 29379648 PMC5759713

[24] Newall P Moodie C Reith G Gambling marketing from 2014 to 2018: a literature review. Curr Addict Rep 2019, 10.1007/s40429-019-00239-1 .

[25] Responsible Gambling Strategy Board. The Responsible Gambling Strategy Board’s advice on the National Strategy to Reduce Gambling Harms 2019-2022. https://www.gamblingcommission.gov.uk/PDF/The-Responsible-Gambling-Strategy-Boards-advice.pdf..

[26] West R Tobacco smoking: health impacts, correlates, prevalence and interventions. Psychol Health 2017;32:1018-36. 10.1080/08870446.2017.1325890 28553727 PMC5490618

[27] Abbott M, Binde P, Hodgins D, Pereira A, Volberg R, Williams RJ. Conceptual framework of harmful gambling: an international collaboration (revised). Ontario Problem Gambling Research Centre, 2015.

[28] New Zealand Ministry of Health Strategy to prevent and minimise gambling-related harm 2016/7 and 2017/8. Ministry of Health, 2016.

[29] GambleAware GambleAware annual review 2017/18. GambleAware, 2018.

[30] Remote Gambling Association. RGA calls for government to introduce a statutory levy. 2017 https://www.rga.eu.com/rga-calls-for-government-to-introduce-a-statutory-levy/.

[31] HMRC. Policy paper: gambling taxes. Remote gaming duty increase. 2018. https://www.gov.uk/government/publications/remote-gaming-duty-increase/gambling-taxes-remote-gaming-duty-increase..

[32] Kaufman A Jones Nielsen JD Bowden-Jones H Barriers to treatment for female problem gamblers: a UK perspective. J Gambl Stud 2017;33:975-91. 10.1007/s10899-016-9663-1 28008550 PMC5579153

[33] Ronzitti S Soldini E Smith N Potenza MN Clerici M Bowden-Jones H Current suicidal ideation in treatment-seeking individuals in the United Kingdom with gambling problems. Addict Behav 2017;74:33-40. 10.1016/j.addbeh.2017.05.032 28570912

[34] NHS. The NHS long term plan. 2019. https://www.longtermplan.nhs.uk/wp-content/uploads/2019/01/nhs-long-term-plan.pdf.

[35] Rogers RD, Wardle H, Sharp C, et al. Framing a public health approach to gambling in Wales: challenges and opportunities. Bangor Univeristy, 2019. https://www.bangor.ac.uk/psychology/research/gambling/gambling-and-health-in-wales

